# Spatiotemporal Impacts of Forest Fires on Mountain Vegetation: A Case Study From Langtang National Park, Nepal Himalaya

**DOI:** 10.1002/ece3.72758

**Published:** 2026-01-19

**Authors:** Shiva Pokhrel, Sudeep Thakuri, Chandra Kanta Subedi, Ripu Mardan Kunwar, Krishna Prasad Sharma, Ram Prasad Chaudhary, Suresh Kumar Ghimire

**Affiliations:** ^1^ Central Department of Botany Tribhuvan University Kirtipur Nepal; ^2^ Research Centre for Applied Science and Technology (RECAST) Tribhuvan University Kirtipur Kirtipur Nepal; ^3^ Central Department of Environmental Science Tribhuvan University Kirtipur Nepal; ^4^ Tri‐Chandra Multiple Campus Tribhuvan University Kathmandu Nepal

**Keywords:** climate, forest fire, Himalaya, Langtang National Park

## Abstract

Forest fires in the Himalayan region are increasing under climate change, yet their interactions with vegetation dynamics and anthropogenic drivers in protected areas remain poorly understood. This study presents a two decade (2000–2020) spatiotemporal assessment of these interactions in Langtang National Park, Nepal. We integrated MODIS‐derived active‐fire and burned‐area data with vegetation indices, including the Normalized Difference Vegetation Index (NDVI) and Vegetation Condition Index (VCI), along with climatic variables (CHIRPS precipitation and land surface temperature, LST). Trends and drivers were validated through field ecological surveys and stakeholder interviews. Our analysis revealed a significant long‐term greening trend (increasing max NDVI; *Z* = 2.2044, *p* = 0.0275) alongside a stable fire regime. Fire activity showed strong land‐cover specificity, disproportionately affecting grasslands and needle‐leaf forests, whereas closed broadleaf forests exhibited high resilience. Maximum temperature was the primary climatic driver of burned‐area extent (*r* = 0.62, *p* < 0.01), and VCI was the strongest predictor of overall vegetation health (*β* = 0.6285, *p* < 0.001). Field evidence confirmed fire‐mediated ecological succession and highlighted intentional burning as a key anthropogenic ignition source. These findings advance understanding of Himalayan fire ecology, demonstrating that climatic warming and land cover interact to shape fire risk even in a greening landscape. We propose a stratified management framework targeting high‐risk zones, leveraging resistant forests as natural firebreaks, carrying out controlled burning in areas where artificial firebreaks can be created, conducting early season burns in key sensitive zones, and incorporating VCI into early warning systems to enhance the resilience of Himalayan‐protected areas.

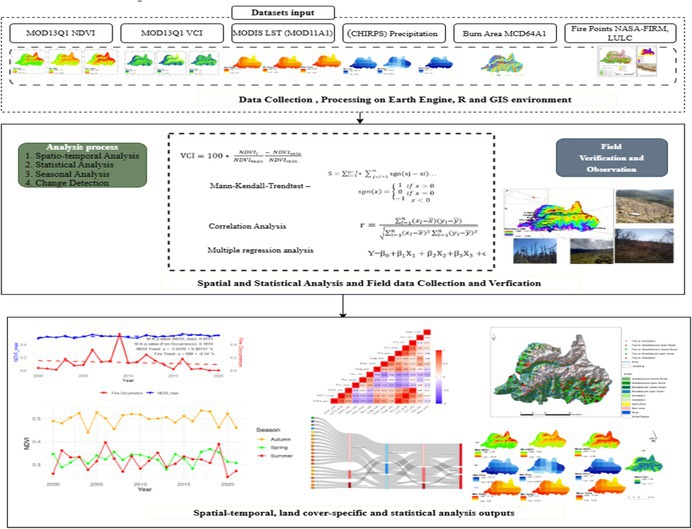

## Graphical abstract

A good positive correlation was found between maximum temperature and burn area; increased temperature relates to an increase in fire activity and larger burn areas. Grassland and needle leaf forests are the major land cover types with relatively high fire frequency, likely due to flammable biomass and seasonal dryness, while broad leaved closed forests displayed better fire resilience.

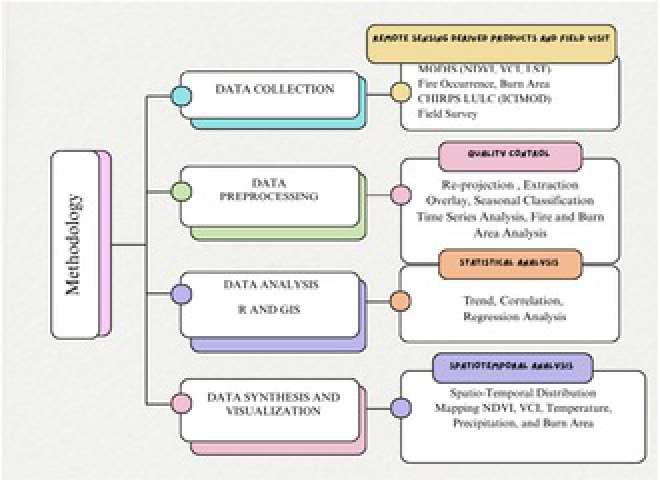

## 1 Introduction

Fire has been a fundamental ecological process and a key tool for landscape management in terrestrial ecosystems for millennia (McLauchlan et al. 2020). Ecologically, fire plays a critical role in nutrient cycling, soil chemistry, and vegetation succession, thereby maintaining ecosystem dynamics (Rogers et al. 2020). The outcomes of fire are strongly dependent on its regime; while low‐intensity, seasonal fires can enhance biodiversity and nutrient turnover, the increasing frequency and severity of fires, often driven by anthropogenic activities and climate change, can lead to soil degradation, reduced forest resilience, and the disruption of essential ecosystem services (Bussmann 2001; Berlanga‐Robles et al. 2018; Cunningham et al. 2024). Mountainous regions are particularly vulnerable to these disturbances. Their steep topography, diverse microclimates, and often flammable vegetation amplify fire spread and ecological damage (Pokhrel 2018; Joshi et al. 2025). In the Himalayan context, fires contribute to habitat fragmentation, accelerated soil erosion, and landslides, compounding the inherent vulnerability of these fragile ecosystems (Matin et al. 2017). In Nepal, wildfires are predominantly anthropogenic, with local communities historically using fire to promote pasture regeneration, clear agricultural land, facilitate hunting, and collect forest products (Kunwar and Khaling 2006; Paudel et al. 2020; Dahal et al. 2025). This has resulted in widespread fire activity across Nepal's diverse physiographic regions, leading to substantial ecological degradation and far‐reaching socio‐economic consequences. The increasing risk of forest fires in Nepal is driven by a combination of factors, including vegetation composition, anthropogenic pressures, climatic variability, and complex topographical conditions (Matin et al. 2017; Parajuli et al. 2023; Tiwari et al. 2022).

Each year, the socio‐economic impact of fires has been severe, resulting in considerable fatalities and substantial financial losses (Pandey et al. 2022; Bhujel et al. 2022). Fire activity in Nepal exhibits a pronounced seasonal pattern, with nearly 90% of fires occurring during the pre‐monsoon months (February–June), when low humidity, high temperatures, and strong winds create ideal conditions (Hamal et al. 2022; Joshi et al. 2025). Khanal (2015) reported that around 375,000 ha of forest were burnt during the period from 2000 to 2014. This upward trajectory is increasingly linked to climate change, with prolonged droughts and higher temperatures extending the fire season and drying biomass, thereby intensifying fire risks (Mishra et al. 2023; Pokharel et al. 2023; Dahal et al. 2025). The escalating fire threat underscores the urgent need for robust monitoring and assessment systems tailored to mountain environments. Advances in remote sensing provide powerful tools for this purpose. Satellites such as MODIS, Landsat, and Sentinel enable detection of active fires to burned areas estimation (Zhang et al. 2025; Guo et al. 2025).

While multispectral indices like the Normalized Difference Vegetation Index (NDVI) and the Vegetation Condition Index (VCI) are effective for quantifying vegetation health, drought stress, and post‐fire recovery (Fensholt et al. 2009; Li et al. 2019; Bento et al. 2020; Kganyago and Shikwambana 2020; Ali et al. 2021; Kourouma et al. 2021; Ntinopoulos et al. 2023; Nyongesa et al. 2023; Mehmood et al. 2024). Globally, these tools have been extensively used to analyze fire impacts, yet integrated spatiotemporal studies that link fire dynamics, climatic variables, and vegetation responses remain scarce for the protected areas of the Nepal Himalaya, including Langtang National Park (LNP). To address this critical research gap, this study employs an integrated approach combining two decades (2000–2020) of satellite‐derived data with field‐based key informant interviews. We aim to characterize the spatiotemporal patterns of fire occurrences across different land cover types in LNP and assess their impacts on mountain vegetation health using NDVI and VCI. Furthermore, we seek to quantify the relationships between fire activity, key climatic drivers, and vegetation indices, while contextualizing these findings with local stakeholder knowledge to understand anthropogenic influences and perceived impacts. By elucidating the complex interactions between fire, vegetation, and climate in a high‐value conservation area, this research provides critical insights to support adaptive fire management, risk zonation, and ecosystem resilience strategies in the face of a changing climate.

## 2 Data and Methods

### 2.1 Study Area

**FIGURE 1:**
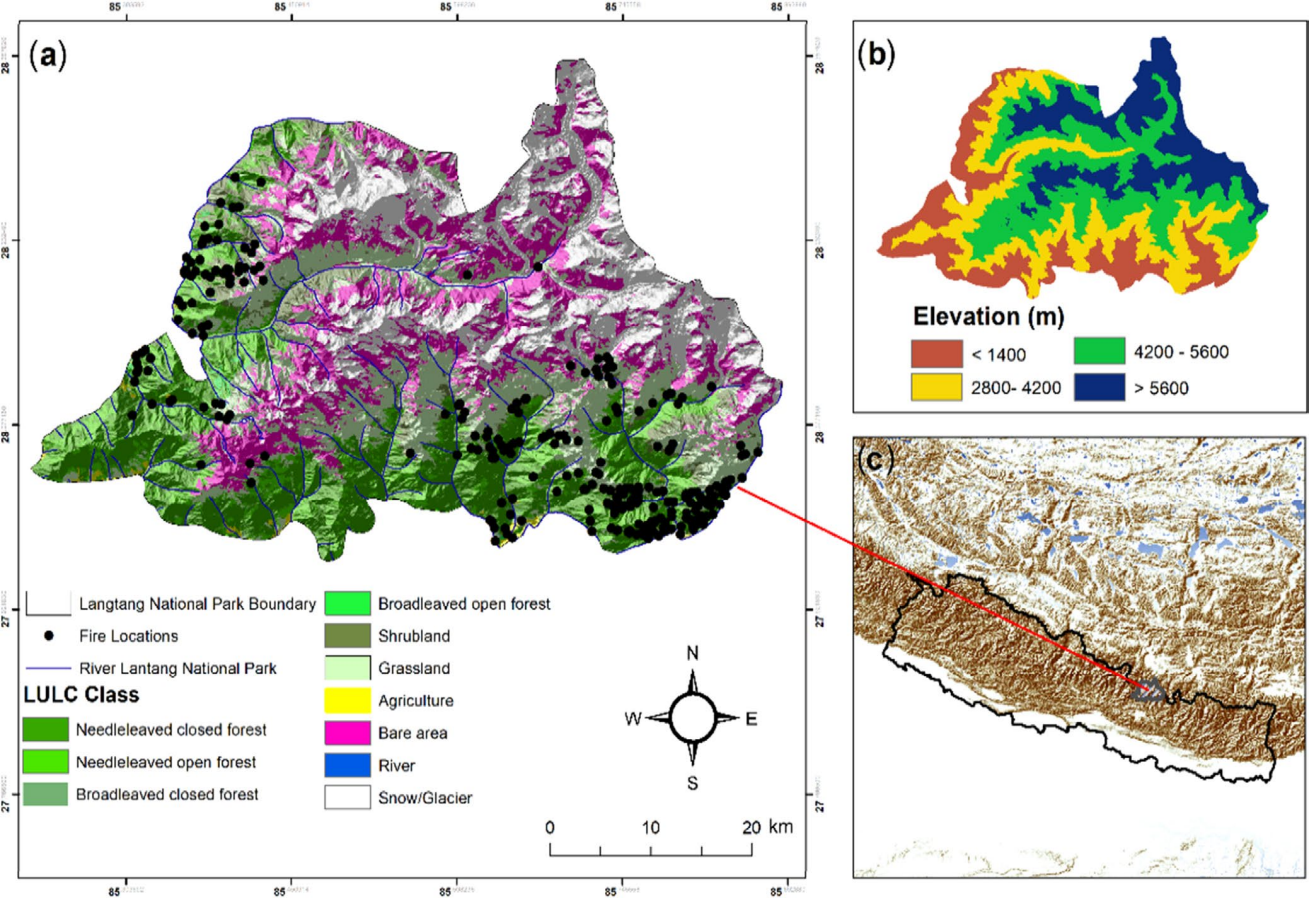
Map illustrating: (a) land use and land cover, including fire locations from 2000 to 2020; (b) elevation distribution of the study area; and (c) the study area's location within Nepal.

LNP was established in 1976, which is the first Himalayan National Park of Nepal (Figure 1). The National park covers an area of 1710 km^2^ including 420 km^2^ buffer zone. The Park covers certain areas of Rasuwa, Nuwakot, and Sindhupalachok districts of the central Himalayan region of Nepal. Geographically, it is located between 28°10′26″ N and 85°33′11″ E with northern and eastern borders to the Qomolangma National Nature Preserve in the Tibet autonomous region of China and in the west, it is bounded by the Bhote Koshi and Trishuli river systems. The park covers the diverse topography and geology with varying climatic conditions ranging from tropical in the south to arctic in the higher elevations in the north. The regions' elevation distribution ranges from 792 to 7245 m above sea level (asl), including Mt. Langtang Lirung (Chaudhary 1998). The park comprises six major climatic zones, namely tropical, subtropical, temperate, subalpine, alpine, and tundra. The southern part of the park experiences a warmer climate with summer temperatures reaching an average of 30°C and winter temperatures around 10°C, while the northern higher elevations remain below freezing.

From December to May, the park experiences the winter with snow cover in various parts of higher elevation areas. While from June to September, the monsoon period brings rainfall with an average annual precipitation ranging from 650 mm at Langtang station (3920 m asl) to 1800 mm at Dhunche station (1950 m asl). The park is rich in biodiversity, home to over 46 species of mammals, 345 bird species, and approximately 1000 vascular plant species, 172 of which are used for medicinal purposes. The spatial resolution of our primary datasets (ranging from 250 to 1 km) presents a consideration for interpreting fine‐grained patterns within the 1710 km^2^ study area. However, the selection of Langtang National Park (LNP) is justified by its role as a biodiversity hotspot and a protected area encapsulating the complete Himalayan ecological gradient. This makes it an ideal natural laboratory for studying mountain fire dynamics. To ensure analytical robustness, our study design prioritized the analysis of *dominant spatiotemporal trends* across the park. We supplemented coarser‐resolution data (1 km LST and fire occurrences) with finer‐resolution inputs (250 m NDVI/VCI, 30 m LULC) to enhance pattern detection. Furthermore, all trend and correlation analyses were performed on park‐wide aggregated time series or across major land cover classes, an approach that is well‐suited to the data resolution and effectively characterizes the overarching system dynamics. The specific implications of scale for our findings are discussed in the limitations section.

### 2.2 Vegetation in the Langtang National Park

The LNP is home to a remarkable diversity of flora, including 15 flowering plant species unique to the region, such as *Rhododendron cowanianum*, *R*. *lowndesii*, and *Larix potaninii* var. *himalaica*, among others. Based on the bioclimatic zones, there are 18 types of forest in the national park. In the tropical zone (below 1000 m), forests are dominated by Sal ( *Shorea robusta* ), limited to the lower Bhote Koshi Khola and Trishuli River areas. The subtropical zone (1000–2000 m) features *Schima wallichii* and *Castanopsis indica* in moist areas, with *Pinus roxburghii* occupying drier slopes. Shrubs like *Berberis aristata* and herbs, including *Artemisia vulgaris* , are common in heavily grazed areas (Chaudhary 1998). The temperate zone (2000–3000 m) supports oak forests (*Quercus* spp.), *Rhododendron arboreum*, and species such as *Ilex dipyrena* and *Lyonia ovalifolia*. In drier areas, *Pinus wallichiana* predominates, while *Tsuga dumosa* and *Picea smithiana* thrive in damp, shaded zones. Bamboo thickets are also widespread. The subalpine zone (3000–4000 m) is characterized by conifers like *Tsuga dumosa*, *Abies spectabilis*, and *Larix potaninii* var. *himalaica* in the lower regions. Near the treeline (3900–4000 m), *Betula utilis* and *Rhododendron campanulatum* are prevalent. Shrubs such as *Juniperus recurva*, *Ephedra gerardiana* , and *Rhododendron anthopogon* dominate the upper subalpine areas. In the alpine zone (above 4000 m), vegetation transitions to shrubs like *Rhododendron nivale* and *R. anthopogon*, giving way to alpine meadows filled with herbs such as different species of *Primula* and *Potentilla* in the upper reaches (4500–5000 m). The National Park is home to diverse endemic species and provides safe, suitable habitats for those species. Plant species like *Liparis langtangensis*, *Meconopsis autumnalis*, *Berberis orthobotrys*, *Lalldhwojia pastinacifolia*, and *Oreocome involucellate* are endemic to the region, and these resources have significant ecological value, as they support habitats for wildlife and provide resources for the local communities (Chaudhary 1998; LNP 2020). Recent studies on high‐altitudinal vegetation dynamics, including treeline ecotones, further underscore the park's ecological complexity and sensitivity to environmental change (Baniya et al. 2021).

### 2.3 Data Sources and Preprocessing

This study utilizes multi‐source satellite datasets spanning 2000–2020 to analyze fire‐vegetation‐climate interactions in Langtang National Park. The datasets include vegetation indices, climate variables, fire occurrences, and land cover information, as detailed in Table 1. All data remote sensing derived data processing was conducted using Google Earth Engine (GEE) to ensure computational efficiency and reproducibility. Fire occurrence data were downloaded from NASA's FIRMS (MODIS Collection 6.1), and only detections with a *high* confidence level were selected for analysis to ensure data reliability and minimize false positives.

**TABLE 1:** Data types, sources, and resolution.

Types (input variables)	Source	Unit/Resolution	References
Normalized Difference Vegetation Index (NDVI)	MOD13Q1 www.modis.gsfc.nasa.gov	250 m	Justice et al. (2002), Tucker et al. (2005), Didan (2015)
Vegetation Condition Index (VCI)	MOD13Q1 www.modis.gsfc.nasa.gov	250 m	Jiao et al. (2016)
Land Surface Temperature (LST)	MODIS LST (MOD11A1) www.lpdaac.usgs.gov	1 km	
Climate Hazards Group InfraRed Precipitation with Station data (CHIRPS)	www.chc.ucsb.edu/data/chirps	0.05 degree	
Burn Area (BA)	MCD64A1 www.lpdaac.usgs.gov	500 m	
Land Use and Land Cover (LULC) dataset	www.rds.icimod.org	30 m	
Fire Occurrence	firms.modaps.eosdis.nasa.gov	1 km	

This quality filtering process, based on the product's quality assurance flags, is particularly important for reducing commission errors in complex mountainous terrain, where cloud contamination and mixed‐pixel effects can otherwise lead to inaccurate fire detection. The MODIS active fire data were supplemented with the MCD64A1 burned area product, which provides comprehensive spatial coverage of fire‐affected areas, though with noted limitations in detecting small fires. For vegetation monitoring, we employed MODIS‐derived NDVI (MOD13Q1) at 250 m resolution and calculated the Vegetation Condition Index (VCI) to assess moisture‐related vegetation stress with Equation (1). Climate data included Land Surface Temperature from MODIS (MOD11A1) and precipitation estimates from the Climate Hazards Group InfraRed Precipitation with Station data (CHIRPS). The high‐resolution (30 m) land use and land cover data were obtained from ICIMOD, providing detailed classification of vegetation types essential for analyzing fire distribution across different land cover classes.

The data detailed with various metadata and sources are shown in Table 1.

(1) $$\mathrm{VCI}=\frac{100^{*}{\text{NDVI}}_{i}}{{\text{NDVI}}_{\max}}\frac{-{\text{NDVI}}_{\min}}{{\text{NDVI}}_{\min}}$$

here, NDVI_*i*_ represents the monthly NDVI values for each pixel, which is calculated from 7 day composite images for a given month such as February; NDVI_min_ indicates minimum NDVI value observed for each pixel during the study period (2000–2020); NDVI_max_ Maximum represents NDVI value observed during the study period (2000–2020).

Since the VCI estimated values indicate the vegetation conditions by comparing the actual NDVI to the historical range, the greater the VCI values, the better the vegetation.

### 2.4 Methods

**FIGURE 2:**
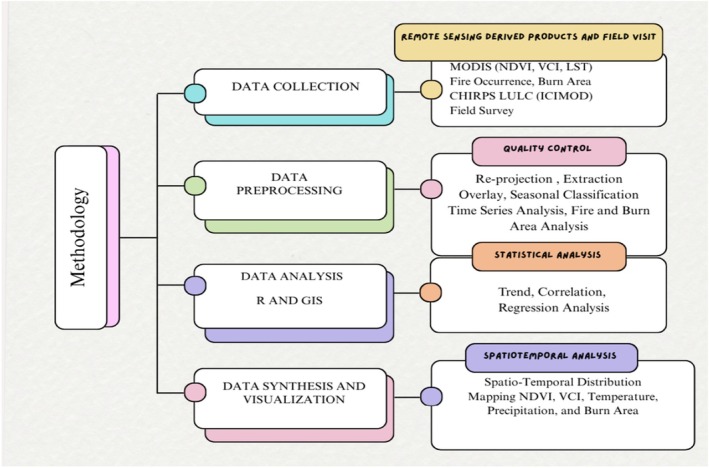
Methodological flow diagram.

The Figure 2 shows the methodological framework used for this study. The study assesses several parameters, namely maximum, mean, and minimum monthly, annual NDVI, VCI, precipitation, LST, and annual burn area over the study period. Vegetation data including the land use and land cover data were obtained from ICIMOD. Similarly, the growing period was calculated, considering May to October during which vegetation experiences growth and development. The period is followed by the fire season in the study area, allowing us to examine how NDVI and VCI behave during the optimal growing season. This period follows the fire season in the study area, allowing us to examine how NDVI and VCI behave during optimal growing conditions. To better understand the cause and impacts of fire as well as to validate the remote sensing derived data, field work and key informant interviews were carried out with hotel owner's restaurant operators, yak herders and local potters in Deurali, Chandanbari, and Laurebina within LNP.

### 2.5 Statistical Framework for Trend and Relationship Analysis

To quantify long‐term trends and relationships between fire activity, climate, and vegetation health, we applied a suite of statistical analyses to park‐wide aggregated data (2000–2020). First, the non‐parametric Mann–Kendall trend test (Equation 2) was employed to detect significant monotonic trends. In this test, the test statistic S indicates the direction of the trend (positive for increasing, negative for decreasing), from which the normalized *Z*‐score and Kendall's tau (*τ*), a measure of trend strength, are derived (Nyeko‐Ogiramoi et al. 2013). Second, Pearson's correlation analysis (Equation 3) was used to quantify linear relationships. The correlation coefficient *r* measures the strength and direction of this linear relationship, ranging from −1 to +1 (Mo et al. 2019). Finally, a multiple linear regression model (Equation 4) was developed to evaluate the combined influence of predictors. In this model, Y represents the dependent variable (e.g., Mean NDVI), which is expressed as a function of several independent variables weighted by their regression coefficients (*β*), which quantify their individual impact. All statistical computations were performed using R Language version 4.0.1 (R Core Team 2024).

(2) $$S=\sum_{i=1}^{n-1}\sum_{j=i+1}^{n}\mathrm{sgn}\left(\mathrm{xj}-\mathrm{xi}\right)$$

$$\mathrm{sgn}\left(x\right)=\left\{\begin{array}{cc} 1 & \text{if}\;x>0\\ 0 & \text{if}\;x=0\\ -1 & x<0 \end{array}\right.$$

(3) $$r=\frac{\sum_{i=1}^{n}\left(x_{i}-\bar{x}\right)\left(y_{i}-\bar{y}\right)}{\sqrt{\sum_{i=1}^{n}{\left(x_{i}-\bar{x}\right)}^{2}\sum_{i=1}^{n}{\left(y_{i}-\bar{y}\right)}^{2}}}$$

where $x_{i}$: Data points for the independent variable (e.g., Burn area or Fire occurrence); $\bar{x}$: Mean of $x_{i}$; $y_{i}$: Data points for the dependent variable (Mean NDVI); $\bar{y}$: Mean of $y_{i}$; *n*: Number of observations.
(4)

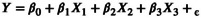

where *Y* represents the dependent variable (Mean NDVI); $X_{1},X_{2}X_{3}$ Represent Fire occurrence, Burn area, and precipitation, respectively; β0,β1,β2^β3: Are the regression coefficients. And ϵ: is the error term.

## 3 Result

### 3.1 Spatiotemporal Patterns of Environmental Variables

**FIGURE 3:**
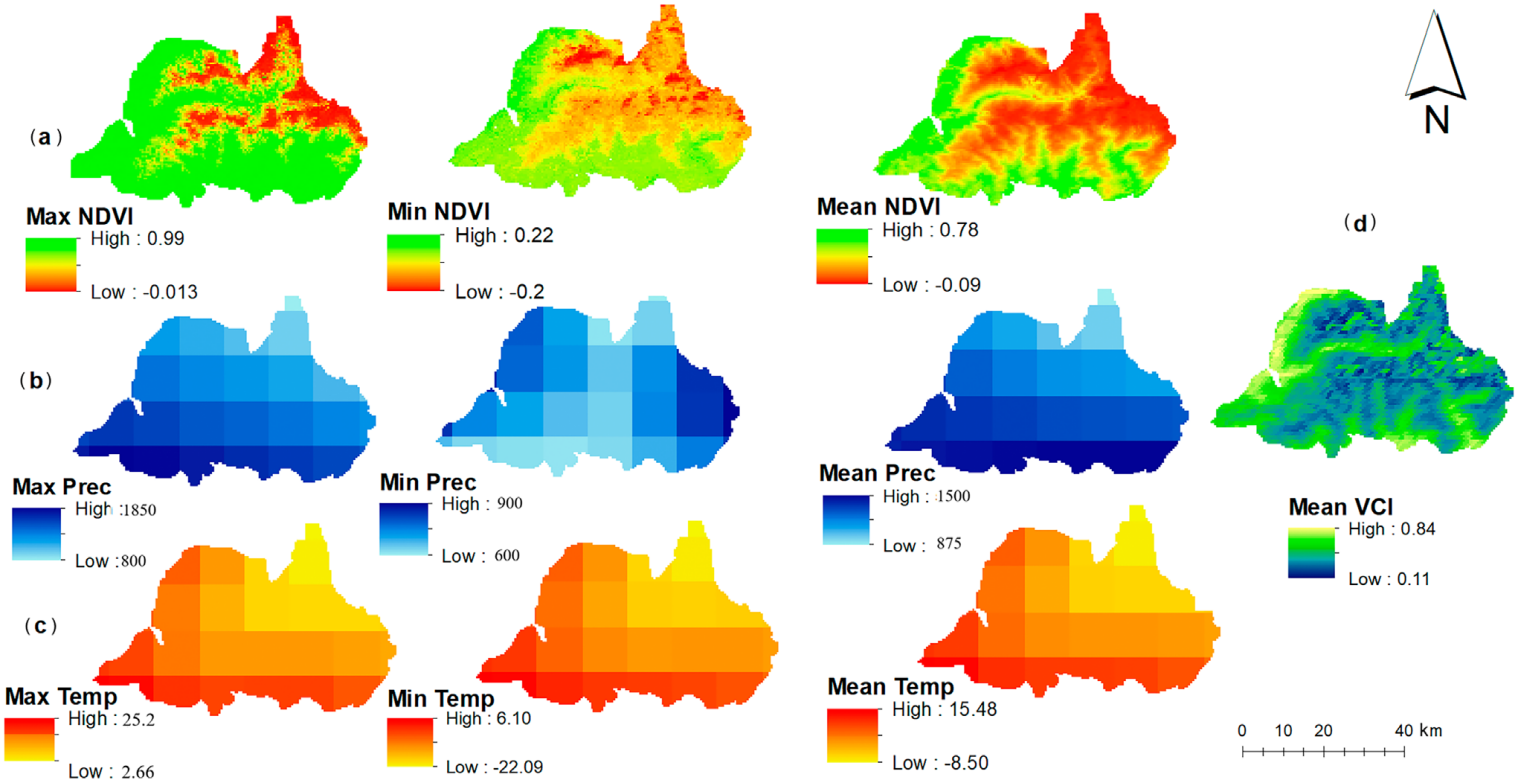
Spatio‐temporal dynamics of (a) NDVI, (b) Precipitation, (c) LST, and (d) Mean VCI from 2000 to 2020.

**FIGURE 4:**
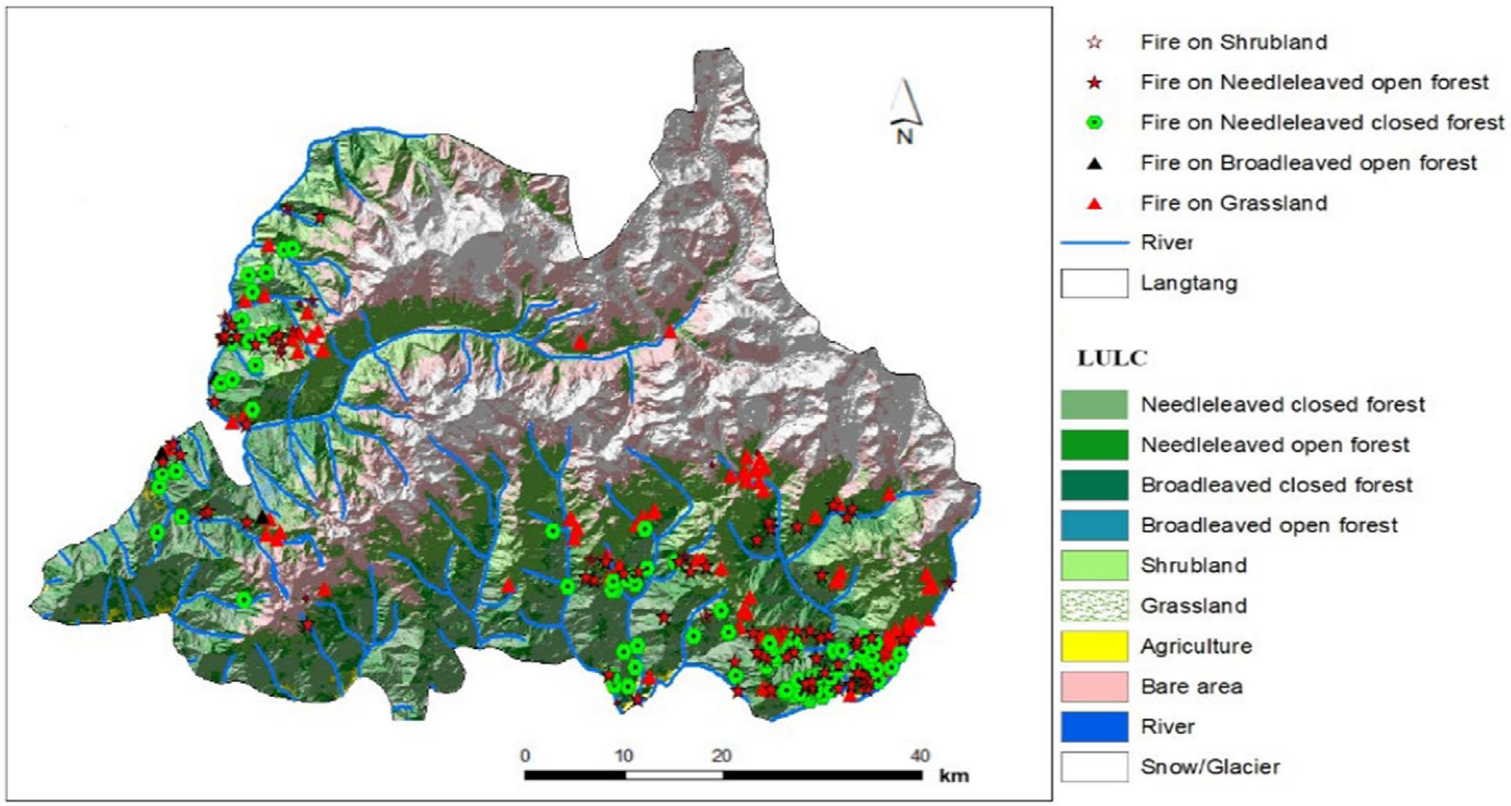
Map showing spatial distribution of fire by vegetation cover.

Analysis of satellite data from 2000 to 2020 revealed distinct environmental gradients across Langtang National Park (LNP). Vegetation density, as measured by NDVI, exhibited a strong altitudinal gradient (Figure 3a). The highest NDVI values (> 0.7) were associated with broadleaved closed forests in the southern lowlands, while needle‐leaved forests at mid‐elevations showed moderate values (0.3–0.5). The alpine zones (above 4000 m) exhibited the lowest NDVI values (< 0.2), indicating sparse vegetation cover. Climatic variables showed complementary patterns. Precipitation was highest (> 1800 mm/year) in the southern parts of the park and decreased significantly to less than 800 mm/year in the northern rain‐shadow regions (Figure 3b). Land Surface Temperature (LST) was highest (> 25°C) in the southern low‐elevation areas and decreased with altitude, though some central valleys showed localized anomalies suggesting microclimatic effects (Figure 3c). The spatial overlay of the 270 high‐confidence fire events showed that fires were predominantly concentrated in the northwestern and southwestern sectors of the park (Figure 4). These areas correspond to zones dominated by grasslands and needle‐leaved forests, linking initial fire risk to specific land cover types.

### 3.2 Spatiotemporal Distribution of Fires by Land Cover Types

Fire distribution varied substantially across different land cover types over the 20‐year period. Grasslands experienced the highest fire frequency, with 97 recorded events, followed by open needle‐leaved forests (83 events) and closed needle‐leaved forests (68 events). Shrublands showed moderate vulnerability (12 events), while broadleaved open forests exhibited greater resilience (8 events). Notably, closed broadleaved forests recorded zero fire events throughout the entire study period, highlighting their strong fire resistance. Temporal analysis revealed significant interannual variability in fire counts, with prominent peaks in 2006 (34 events) and 2009 (60 events). A sharp decline was observed in later years, with minimal activity in 2018 and a complete absence of detected fires in 2019–2020.

### 3.3 Growing Season Vegetation Dynamics and Trends

**FIGURE 5:**
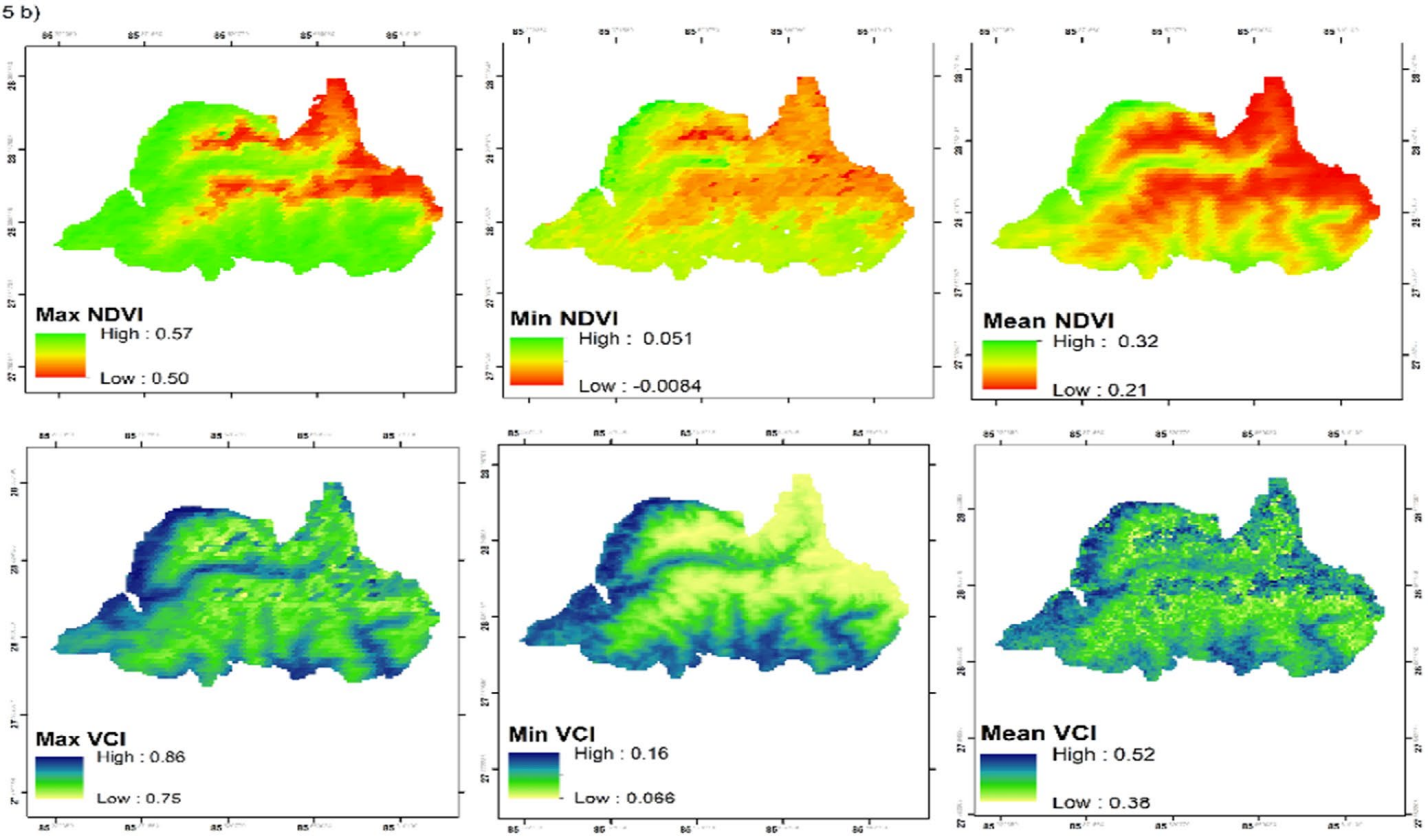
Panels (a) and (b) show the growing season (May–October) distribution of VCI and NDVI from 2000 to 2020.

**FIGURE 6:**
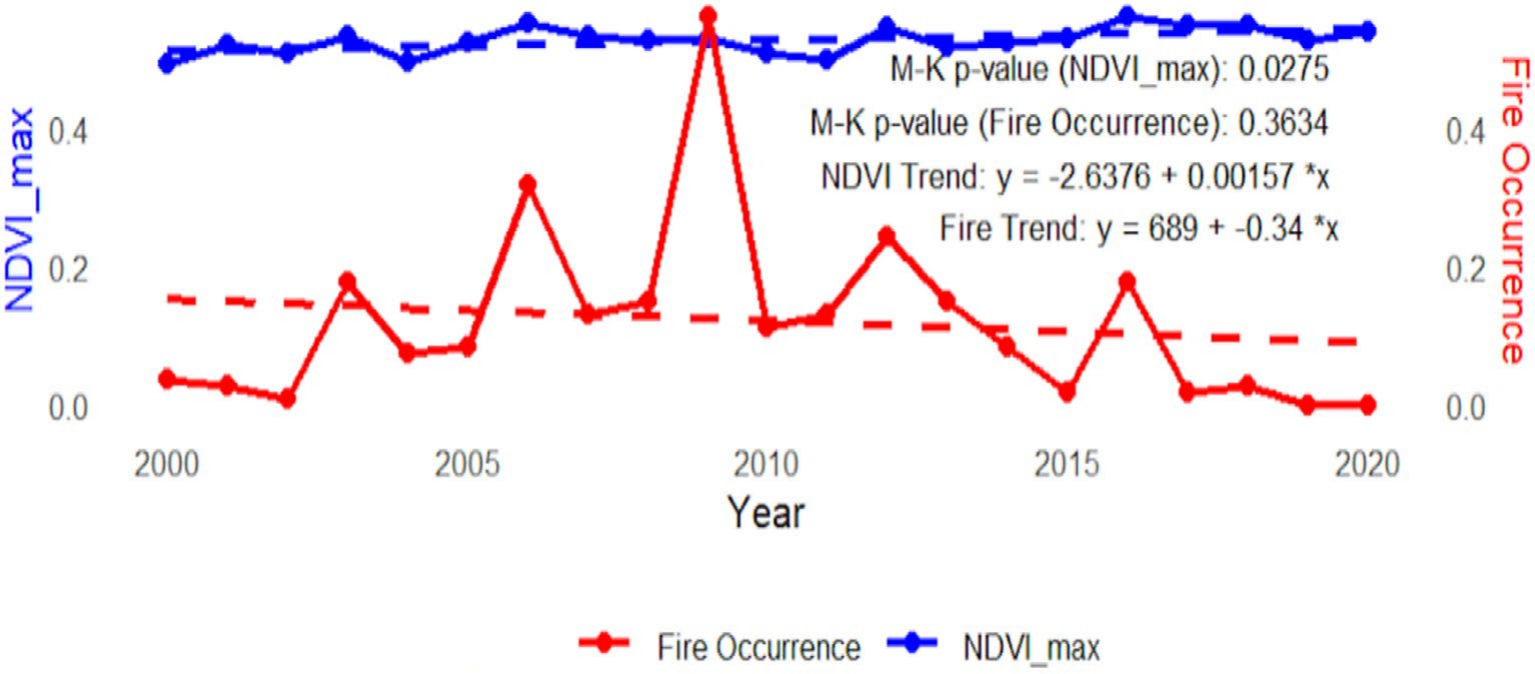
MK trend analysis of NDVI maximum and fire occurrence.

Analysis of the primary growing season (May–October) revealed substantial inter‐annual variability in vegetation health (Figure 5). Maximum NDVI values fluctuated, ranging from a low of 0.493 in 2000 to a peak of 0.561 in 2016, indicating optimal vegetation conditions in the latter year. The Vegetation Condition Index (VCI) showed parallel trends, with its maximum value peaking at 0.85 in 2016 and its minimum plummeting to 0.065 in 2020, reflecting pronounced vegetation stress. The Mann–Kendall trend test revealed a statistically significant positive trend in annual maximum NDVI over the study period (Z = 2.2044, *p* = 0.0275), indicating a general “greening” trend across LNP (Table 2, Figure 6). In contrast, the trend in fire occurrence was not significant (*p* = 0.3634).

**TABLE 2:** Mann–Kendall (MK) trend test results.

Test parameter	NDVI_max	Fire occurrence
S	74.00	−31.00
Variance (varS)	1096.67	1089.67
Tau (*τ*)	0.352381	−0.150143
Z‐Score	2.2044	−0.9088
*p*	0.0275*	0.3634

* Significant at the 95% confidence level (*p* < 0.05).

### 3.4 Correlation Analysis

**FIGURE 7:**
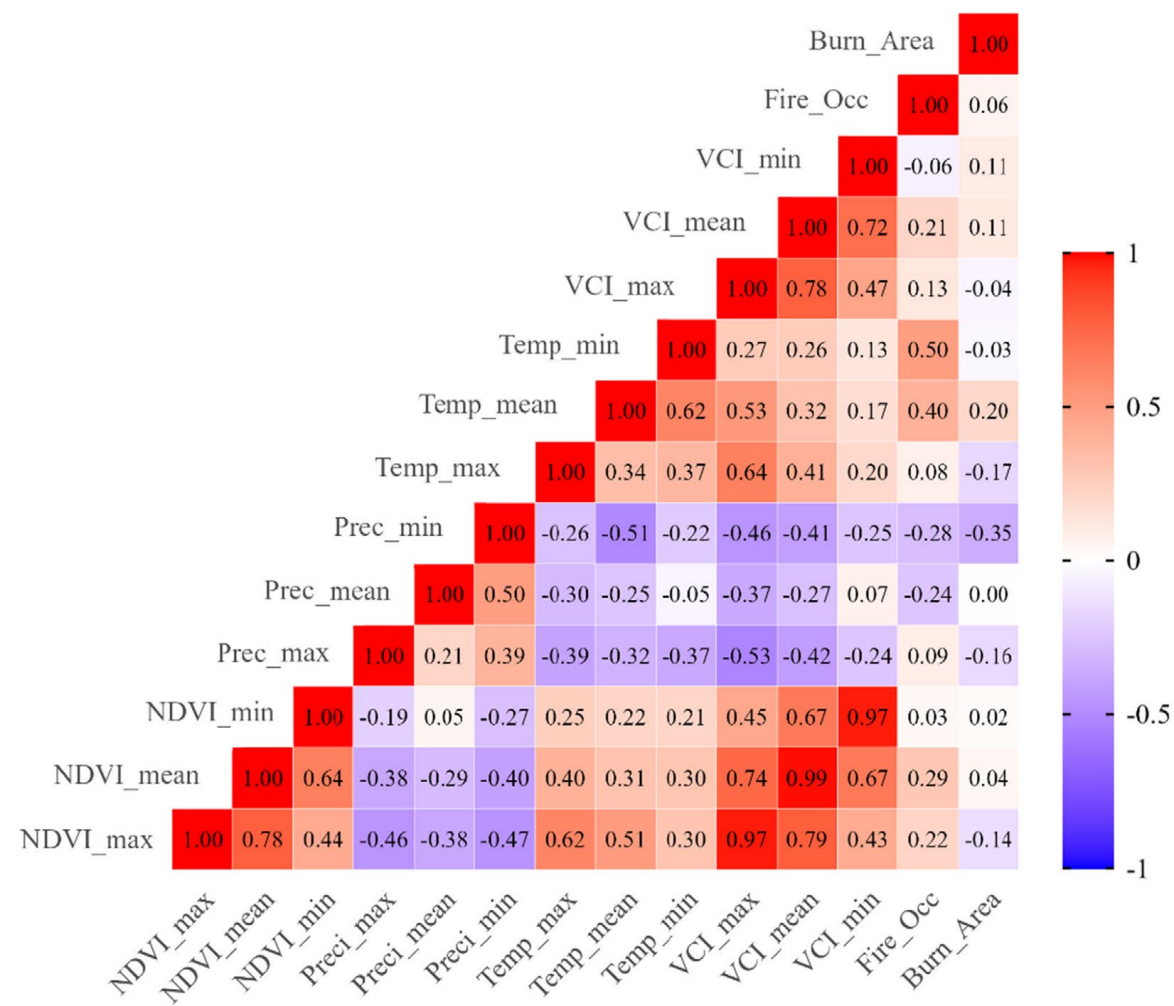
Correlation analysis among variables.

Correlation analysis among environmental variables revealed several significant relationships (Figure 7). A near‐perfect positive correlation was found between NDVI mean and VCI mean (*r* = 0.99, *p* < 0.001). Maximum temperature showed a strong positive correlation with burn area (*r* = 0.62, *p* < 0.01), while minimum precipitation correlated negatively with burn area (*r* = −0.47, *p* < 0.05). Fire occurrence demonstrated a moderate positive correlation with minimum temperature (*r* = 0.50, *p* < 0.05), and a weak negative correlation with NDVI maximum (*r* = −0.14, *p* < 0.1). A moderate negative correlation was observed between maximum precipitation and minimum NDVI (*r* = −0.46, *p* < 0.05).

### 3.5 Regression Analysis of NDVI Drivers

**FIGURE 8:**
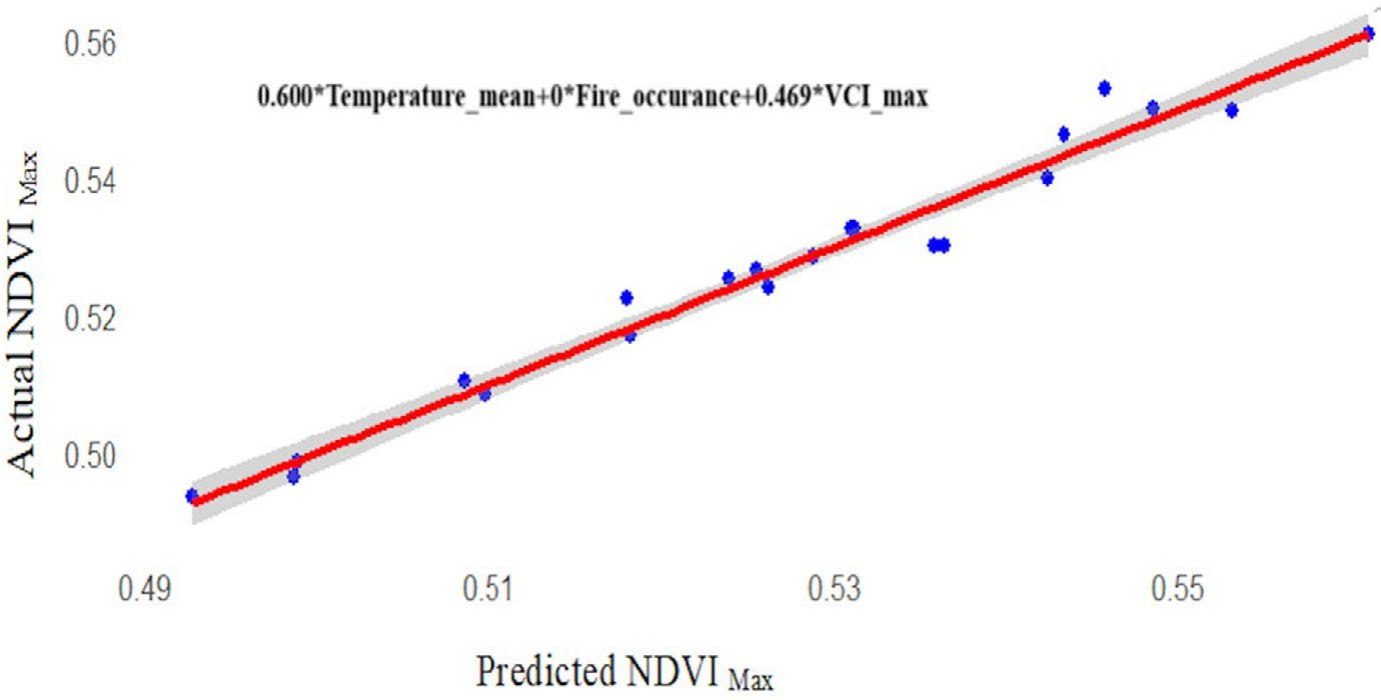
Linear regression analysis between actual and Predicated NDVI_maximum_.

Multiple linear regression explained 95.13% of variance in NDVI maximum (Table 3). VCI maximum was the strongest predictor (*β* = 0.6285, *p* < 0.001), while fire occurrence showed a small but statistically significant positive effect (*β* = 0.000158, *p* = 0.0488). Precipitation mean and temperature mean were not significant predictors in the model (*p* > 0.05). The relationship between actual and predicted NDVI maximum is shown in Figure 8.

**TABLE 3:** Regression analysis.

Variable	Estimate	Std. Error	*t*‐value	*p*
Intercept	0.02602	0.03352	0.776	0.4489
Precipitation mean	0.0002	0.00234	0.086	0.9328
Temperature mean	−0.00272	0.00289	−0.942	0.3604
Fire occurrence	0.000158	0.000074	2.133	0.0488*
VCI_max	0.6285	0.03887	16.169	< 0.001***

* *p* < 0.05 statistically significant.

*** *p* < 0.001 very highly significant

### 3.6 Annual Burn Area Patterns

**FIGURE 9:**
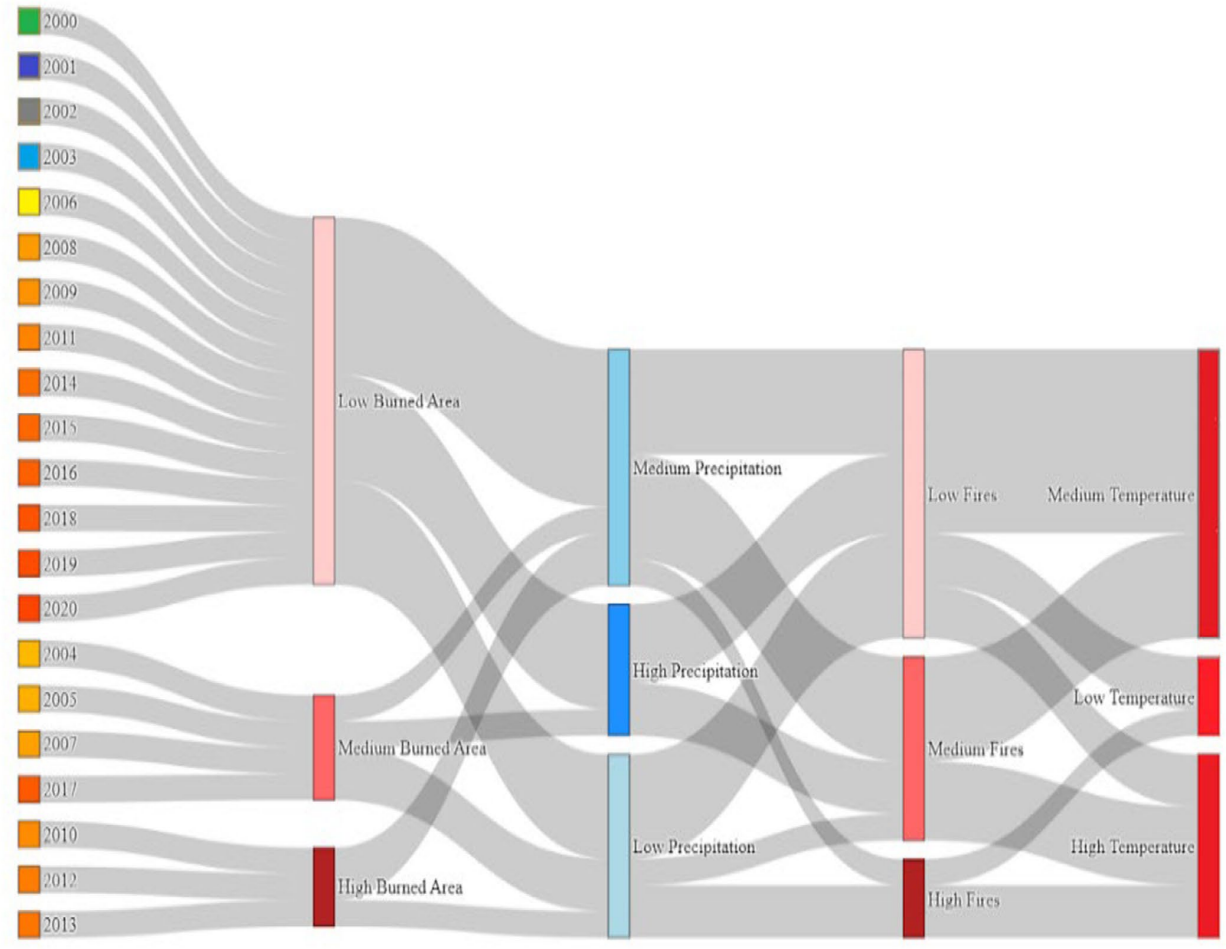
Diagram showing burn area in relation to fire occurrence, precipitation and temperature.

Analysis of burn area in relation to climate drivers showed distinct annual patterns (Figure 9). The highest burn areas occurred in 2010, 2012, and 2013, coinciding with periods of high temperature and low precipitation. Conversely, years with high precipitation (2015, 2018) showed minimal burn area despite moderate temperatures. In 2010, extensive burning occurred with moderate fire occurrence but the highest recorded temperatures.

## 4 Field Data Collection and Stakeholder Analysis

To validate remote sensing observations and understand the socio‐ecological context of fires, we conducted field surveys in May 2024 and September 2024. Data were collected through two primary methods: semi‐structured interviews and direct ecological observation. Stakeholder Interviews: We conducted semi‐structured interviews with 16 key informants from sectors with extensive local environmental knowledge, hotel owners (*n* = 6), yak and goat herders, and porters (*n* = 5 each). A semi‐structured questionnaire was used to ensure consistent coverage of key topics while allowing for open‐ended discussion. The interviews focused on: (1) local knowledge of historical fire locations and causes; (2) perceived ecological and economic impacts of fires; and (3) community reliance on forest resources. This approach of integrating local stakeholder knowledge is recognized as a critical component for comprehensive forest fire assessment.

### 4.1 Ecological Validation

Field visits were made to four fire‐impacted sites identified by both satellite data and local respondents. At these sites, we documented post‐fire ecological conditions, including soil characteristics, tree mortality, and vegetation succession patterns. We also quantified resource use by estimating fuelwood collection volumes based on direct observation and local reports.

### 4.2 Perceived Causes and Drivers of Fire

Analysis of interview data revealed distinct patterns in the perceived causes of fire. A significant finding was the divergent perspectives on ignition sources. Yak herders predominantly attributed fires to external actors, such as travelers and poachers. In contrast, hotel owners reported that fires in upper grasslands were often set intentionally to stimulate the growth of fresh grass for grazing livestock. This practice, though its ecological impact was not quantitatively verified by respondents, highlights a recognized anthropogenic driver where fire is used as a land management tool. Other identified ignition causes included hunting, collection of non‐timber forest products, and fuelwood gathering.

### 4.3 Post‐Fire Ecological Shifts and Resource Use

**FIGURE 10:**
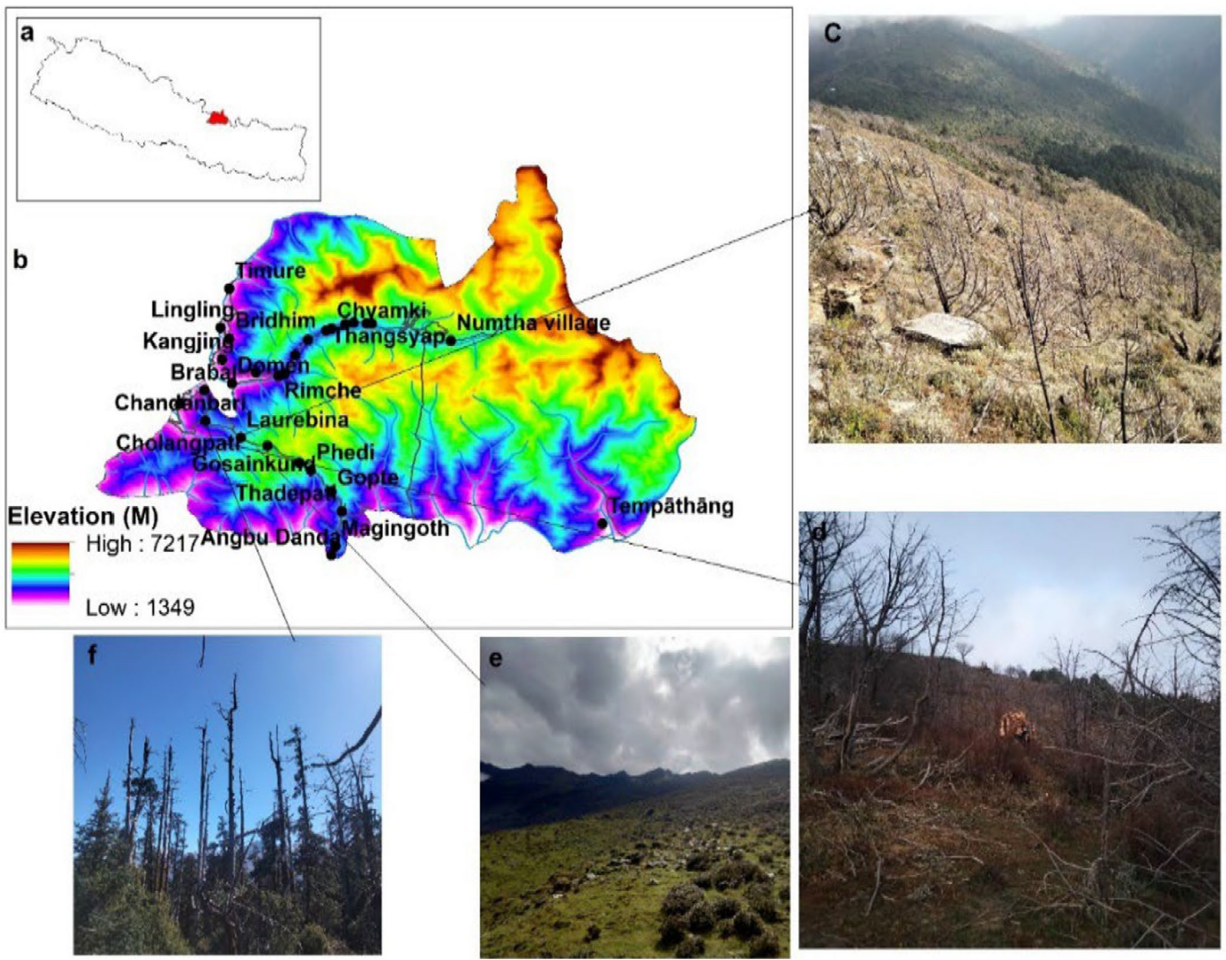
(a) Study area within Nepal, (b) Places and elevation in Langtang National Park (LNP), (c) Forest affected by fire in Cholangpati, (d) Collection of fallen burnt tree wood, (e) Disturbed and burnt grassland with yak grazing, (f) Burnt standing tree in a forest near Chandanbari. Photos © Shiva Pokhrel.

Field observations confirmed significant fire‐induced ecological changes. Ecological documentation showed substantial dead wood accumulation at Cholangpati and Chandanbari, with local communities collecting approximately 90–160 kg of wood daily (3–4 bamboo baskets) from burned areas, demonstrating significant resource dependence. Post‐fire ecological assessments revealed pronounced vegetation transformations, particularly in Cholangpati where a 2019 fire affected hectares of forest, characterized by standing dead trees, blackened soils, and scorched tree trunks (Figure 10c,f). Locals reported that fire was followed by the occurrence and exponential increase of the growth of *Piptanthus nepalensis*.

In another case, after the fire also showed exponential increase in growth with the peak being 1 year later. Where there was no fire, common species found were *Ainsliaea aptera*, *Rubia manjith*, *Viola biflora* , *Circaea alpina* , and *Pilea* sp. in Chandanbari forest whereas the burned‐up areas had dominance by *Senecio diversifolius*, *Anaphalis contorta*, *Drymocallis rupestris*, *Elsholtzia strobilifera*, *Argentina linilaciniata*, *Artemisia roxburghiana*, *Anemonastrum obtusilobum*, *Carex atrata* , and *Gentiana depressa*. In Cholangpati, the common plants in unburned areas were *Anaphalis royleana*, *Ainsliaea aptera*, *Viola biflora* , *Carex atrata* , *Hydrocotyle himalaica*, *Sinocarum* sp., and *Tectaria* sp. The common species in burned areas were *Potentilla heptaphylla* subsp. *heptaphylla*, *Elsholtzia strobilifera*, *Carex atrata* , and *Arenaria* sp.

## 5 Discussion

This study provides a comprehensive analysis of the spatiotemporal interactions between forest fires, vegetation health, and climatic drivers in Langtang National Park (LNP), revealing a complex interplay of climatic forcing, anthropogenic ignition, and ecological resilience. The pronounced concentration of fire events in grasslands and needle‐leaved forests underscores the role of fuel characteristics and land use, with grassland fires reflecting traditional pastoral burning practices (Rawat 1998; Xiong et al. 2020) and needle‐leaved forest susceptibility attributable to flammable resins in coniferous species. The complete absence of fires in closed broadleaved forests highlights their fire‐resistant nature due to dense canopy structure and mesic litter (Peris‐Llopis et al. 2024). Climate emerges as a primary regulator, with the strong positive correlation between maximum temperature and burn area (*r* = 0.62) consistent with global patterns of temperature‐driven fire spread (Westerling et al. 2006; Jolly et al. 2015), while the negative correlation between precipitation and burn area (*r* = −0.47) reaffirms moisture limitation principles in fire ecology (Littell et al. 2016). The significant greening trend indicated by the positive Mann–Kendall trend in maximum NDVI (Z = 2.2044, *p* = 0.0275) suggests overall ecosystem resilience potentially reflecting climate change or conservation policy (Baniya et al. 2021; Pokhrel and Sherpa 2025), though the weak negative correlation between fire occurrence and NDVI maximum (*r* = −0.14) indicates fire acts as a localized stressor.

The parallel behavior of NDVI and VCI during growing seasons in Nepal confirms their reliability as co‐indicators of vegetation health. In our study, peak values during favorable years (e.g., 2006, 2012, 2016) and declines during stress periods (e.g., 2000, 2020) highlight the overarching influence of climate on vegetation dynamics. This observation aligns with previous findings of significant long‐term greening trends across Nepal from the 1980s to the 2020s (Krakauer et al. 2017; Tang and Leng 2022). Interannual fluctuations in NDVI have been largely attributed to variations in temperature and precipitation, which modulate vegetation productivity and stress responses (Baniya et al. 2018, 2019). Our results emphasize the sensitivity of vegetation to drought and moisture variability, particularly in regions with limited water availability, reinforcing the utility of NDVI and VCI as indicators for monitoring ecosystem health and environmental stress (Shrestha et al. 2024; Sanz et al. 2024). These indices thus provide critical insights for understanding vegetation‐climate interactions and for informing management strategies in Nepal's heterogeneous landscapes.

Field evidence corroborates fire‐induced ecological transformations, with successional shifts from mature forest species (e.g., *Ainsliaea aptera*, *Viola biflora* ) to pioneer species (e.g., *Piptanthus nepalensis*, *Senecio diversifolius*) in burned areas, while substantial fuelwood collection (90–160 kg/day) demonstrates local hotel operators' reliance on fire‐affected resources and seems beneficiary of those post fire resources. The divergent perceptions of fire causation between yak herders, often attributing fires to external actors, and hotel owners, who report intentional burning but differ by location, highlight complex socio‐ecological dynamics that require inclusive community engagement. In the Langtang region, forests located closer to human settlements suffered heavier degradation, as the intensity of biomass extraction and other human disturbances tends to increase with proximity to communities (Måren and Sharma 2018). Regression analysis confirms that VCI maximum is the dominant predictor of NDVI maximum (*β* = 0.6285, *p* < 0.001), underscoring its utility for vegetation monitoring (Jiao et al. 2016). Stratified fire patterns further advocate for targeted management, prioritizing high‐risk grasslands and needle‐leaved forests while utilizing closed broadleaved forests as natural firebreaks. Although MODIS spatial resolution limitations may affect small fire detection (Giglio et al. 2013), our integrated approach provides critical insights for developing adaptive fire management strategies in Himalayan protected areas under changing climate conditions.

## 6 Conclusion

This study delivers a comprehensive, multi‐decade assessment of fire‐vegetation‐climate dynamics in Langtang National Park (LNP) by integrating two decades (2000–2020) of remote sensing and field data. Three core findings emerge. First, we document a clear hierarchy of fire susceptibility; activity is concentrated in grasslands and needle‐leaved forests during the dry pre‐monsoon period, demonstrating pronounced land‐cover specificity, a pattern consistent with broader Himalayan fire ecology research. Second, we reveal a critical resilience dynamic; a significant landscape‐scale greening trend (maximum NDVI: Z = 2.2044, *p* = 0.0275) contrasts with the absence of a long‐term rise in fire occurrence, suggesting that, under current regimes, the ecosystem exhibits a capacity for recovery despite localized disturbances. Third, statistical analysis identifies the Vegetation Condition Index (VCI) as the strongest predictor of vegetation health (*β* = 0.6285, *p* < 0.001), confirming its operational utility for monitoring, while climate drivers maximum temperature (*r* = 0.62) and low precipitation (*r* = −0.47) exert dominant control over burn area, aligning with global and regional fire‐climate relationships. Our integrated approach underscores the profound socio‐ecological impacts of fire, from successional shifts toward pioneer species to intensive post‐fire fuelwood extraction by hotels and restaurants inside LNP, highlighting how fire reshapes both ecosystem structure and human resource use.

A transformation that has also been documented in Himalayan forest studies where fire alters species composition and post‐fire community dynamics. While MODIS data limitations constrain small‐fire detection, this long‐term dataset provides a robust foundation for landscape‐scale insight. Future research must incorporate higher‐resolution sensors and additional stressors like snow cover dynamics and soil moisture variability to refine predictive models. These findings directly inform a stratified, climate‐smart fire management framework for LNP. This strategy should: (1) prioritize intervention in high‐risk grasslands and needle‐leaved forests; (2) leverage fire‐resistant broad‐leaved forests as ecological buffers; and implement controlled and early‑season burning where appropriate, including establishment of artificial firebreaks to reduce fire spread; (3) integrate validated indices like VCI into early‐warning systems. By contextualizing our results within the wider Himalayan research context, this work establishes a critical scientific baseline for adaptive, climate‐resilient conservation in the region's vulnerable mountain ecosystems.

## Author Contributions

**Shiva Pokhrel:** data curation (lead), software (lead), validation (lead), visualization (lead), writing – original draft (lead). **Sudeep Thakuri:** investigation (equal), methodology (equal), supervision (equal). **Chandra Kanta Subedi:** methodology (equal), writing – original draft (equal). **Ripu Mardan Kunwar:** conceptualization (equal), writing – review and editing (equal). **Krishna Prasad Sharma:** methodology (equal), validation (equal), writing – review and editing (equal). **Ram Prasad Chaudhary:** supervision (equal), writing – review and editing (equal). **Suresh Kumar Ghimire:** supervision (equal), validation (equal).

## Funding

The study was supported by an innovative research grant from the Research Directorate, Office of the Rector, Tribhuvan University, Nepal. The funders had no role in study design, data collection and analysis, the decision to publish, or preparation of the manuscript.

## Ethics Statement

Permission for this study was obtained from the Department of National Parks and Wildlife Conservation (DNPWC), Nepal.

## Consent

All community participants were informed about the purpose of the research, and verbal consent was obtained prior to their involvement in stakeholder meetings and interviews.

## Conflicts of Interest

The authors declare no conflicts of interest. All ideas expressed in this document do not necessarily represent the views of the organizations/institutions the authors belong to.

## Data Availability

Code used to process and analyze these remote sensing datasets, along with the study area shapefile, are available in the following GitHub repository: https://github.com/greenshia.

## References

[ece372758-bib-0001] Ali, S. , Z. Haixing , M. Qi , et al. 2021. “Monitoring Drought Events and Vegetation Dynamics in Relation to Climate Change Over Mainland China From 1983 to 2016.” Environmental Science and Pollution Research 28, no. 17: 21910–21925. 10.1007/s11356-020-12146-4. 33411304

[ece372758-bib-0002] Baniya, B. , N. P. Gaire , Q. Techato , Y. Dhakal , and Y. P. Dhital . 2021. “High Altitudinal Vegetation Dynamics Including Treeline Ecotone in Langtang National Park, Nepal.” Nepal Journal of Environmental Science 9, no. 2: 13–24. 10.3126/njes.v9i2.36605.

[ece372758-bib-0003] Baniya, B. , Q. Tang , Z. Huang , S. Sun , and K. Techato . 2018. “Spatial and Temporal Variation of NDVI in Response to Climate Change and the Implication for Carbon Dynamics in Nepal.” Forests 9, no. 6: 329. 10.3390/f9060329.

[ece372758-bib-0004] Baniya, B. , Q. Tang , Y. Pokhrel , and X. Xu . 2019. “Vegetation Dynamics and Ecosystem Service Values Changes at National and Provincial Scales in Nepal From 2000 to 2017.” Environmental Development 32: 100464. 10.1016/j.envdev.2019.100464.

[ece372758-bib-0005] Bento, V. A. , C. M. Gouveia , C. C. DaCamara , R. Libonati , and I. F. Trigo . 2020. “The Roles of NDVI and Land Surface Temperature When Using the Vegetation Health Index Over Dry Regions.” Global and Planetary Change 190: 103198. 10.1016/j.gloplacha.2020.103198.

[ece372758-bib-0006] Berlanga‐Robles, C. A. , A. Cervantes‐Escobar , and E. M. Figueroa . 2018. “Estacionalidad y tendencias del bosque tropical caducifolio de la cuenca Piaxtla‐Elota‐Quelite y el área protegida Meseta de Cacaxtla, México.” Madera y Bosques 24: e2431576. 10.21829/myb.2018.2431576.

[ece372758-bib-0007] Bhujel, K. B. , R. P. Sapkota , and U. R. Khadka . 2022. “Temporal and Spatial Distribution of Forest Fires and Their Environmental and Socio‐Economic Implications in Nepal.” Journal of Forest and Livelihood 21, no. 1: 1–13. 10.3126/jfl.v21i1.56575.

[ece372758-bib-0008] Bussmann, R. W. 2001. “Succession and Regeneration Patterns of East African Mountain Forests: A Review.” Systematics and Geography of Plants 71, no. 2: 959–974. 10.2307/3668731.

[ece372758-bib-0009] Chaudhary, R. P. 1998. Biodiversity in Nepal: Status and Conservation. Craftsman Press.

[ece372758-bib-0010] Cunningham, C. X. , G. J. Williamson , and D. M. J. S. Bowman . 2024. “Increasing Frequency and Intensity of the Most Extreme Wildfires on Earth.” Nature Ecology & Evolution 8, no. 8: 1420–1425. 10.1038/s41559-024-02452-2. 38914710

[ece372758-bib-0011] Dahal, K. , R. Talchabhadel , P. Pradhan , et al. 2025. “Nepal's Carbon Stock and Biodiversity Are Under Threat From Climate Exacerbated Forest Fires.” Information Geography 1, no. 1: 100003. 10.1016/j.infgeo.2025.100003.

[ece372758-bib-0012] Didan, K. 2015. “MOD13Q1 MODIS/Terra Vegetation Indices 16‐Day L3 Global 250m SIN V006 [Data Set].” NASA EOSDIS Land Processes DAAC. 10.5067/MODIS/MOD13Q1.006.

[ece372758-bib-0013] Fensholt, R. , K. Rasmussen , T. T. Nielsen , and C. Mbow . 2009. “Evaluation of Earth Observation Based Long Term Vegetation Trends—Intercomparing NDVI Time Series Trend Analysis Consistency of Sahel From AVHRR GIMMS, Terra MODIS and SPOT VGT Data.” Remote Sensing of Environment 113, no. 9: 1886–1898. 10.1016/j.rse.2009.04.004.

[ece372758-bib-0014] Giglio, L. , J. T. Randerson , and G. R. van der Werf . 2013. “Analysis of Daily, Monthly, and Annual Burned Area Using the Fourth‐Generation Global Fire Emissions Database (GFED4).” Journal of Geophysical Research: Biogeosciences 118, no. 1: 317–328. 10.1002/jgrg.20042.

[ece372758-bib-0015] Guo, X. , Y. Liu , P. Liu , H. Wang , and W. Wu . 2025. “Monitoring Active Fires in Borneo From Sentinel‐2 MSI Images.” GIScience & Remote Sensing 62, no. 1: 2539551. 10.1080/15481603.2025.2539551.

[ece372758-bib-0016] Hamal, K. , S. Sharma , N. Khadka , et al. 2022. “Assessment of Drought Impacts on Vegetation Dynamics in Nepal Using MODIS Satellite Products.” Remote Sensing 14, no. 18: 4474. 10.3390/rs14184474.

[ece372758-bib-0017] Jiao, W. , L. Zhang , Q. Chang , D. Fu , Y. Cen , and Q. Tong . 2016. “Evaluating an Enhanced Vegetation Condition Index (VCI) Based on VIUPD for Drought Monitoring in the Continental United States.” Remote Sensing 8, no. 3: 224. 10.3390/rs8030224.

[ece372758-bib-0018] Jolly, W. M. , M. A. Cochrane , P. H. Freeborn , et al. 2015. “Climate‐Induced Variations in Global Wildfire Danger From 1979 to 2013.” Nature Communications 6, no. 1: 7537. 10.1038/ncomms8537. PMC4803474 26172867

[ece372758-bib-0019] Joshi, K. P. , S. Giri , D. Kuinkel , et al. 2025. “Forest Fire Dynamics in Nepal: Regional Trends and Socio‐Ecological Drivers.” Trees, Forests and People 21: 100942. 10.1016/j.tfp.2025.100942.

[ece372758-bib-0020] Justice, C. O. , J. R. G. Townshend , E. F. Vermote , et al. 2002. “An Overview of MODIS Land Data Processing and Product Status.” Remote Sensing of Environment 83, no. 1–2: 3–15. 10.1016/S0034-4257(02)00084-6.

[ece372758-bib-0021] Kganyago, M. , and L. Shikwambana . 2020. “Assessment of the Characteristics of Recent Major Wildfires in the USA, Australia and Brazil in 2018–2019 Using Multi‐Source Satellite Products.” Remote Sensing 12, no. 11: 1803. 10.3390/rs12111803.

[ece372758-bib-0022] Khanal, S. 2015. “Wildfire Trends in Nepal Based on MODIS Burnt‐Area Data.” Banko Janakari 25, no. 1: 76–79.

[ece372758-bib-0023] Kourouma, J. M. , E. Eze , E. Negash , et al. 2021. “Assessing the Spatio‐Temporal Variability of NDVI and VCI as Indices of Crops Productivity in Ethiopia: A Remote Sensing Approach.” Geomatics, Natural Hazards and Risk 12, no. 1: 2880–2903. 10.1080/19475705.2021.1976849.

[ece372758-bib-0024] Krakauer, N. Y. , T. Lakhankar , and J. D. Anadon . 2017. “Mapping and Attributing Normalized Difference Vegetation Index Trends for Nepal.” Remote Sensing 9, no. 10: 986. 10.3390/rs9100986.

[ece372758-bib-0025] Kunwar, R. M. , and S. Khaling . 2006. “Forest Fire in the Terai, Nepal: Causes and Community Management Interventions.” International Forest Fire News 34: 46–54.

[ece372758-bib-0026] Li, F. , X. Zhang , D. P. Roy , and S. Kondragunta . 2019. “Estimation of Biomass‐Burning Emissions by Fusing the Fire Radiative Power Retrievals From Polar‐Orbiting and Geostationary Satellites Across the Conterminous United States.” Atmospheric Environment 211: 94–104. 10.1016/j.atmosenv.2019.05.017.

[ece372758-bib-0027] Littell, J. S. , D. L. Peterson , K. L. Riley , Y. Liu , and C. H. Luce . 2016. “A Review of the Relationships Between Drought and Forest Fire in the United States.” Global Change Biology 22, no. 7: 2353–2369. 10.1111/gcb.13275. 27090489

[ece372758-bib-0028] LNP . 2020. Management Plan of Langtang National Park and its Buffer Zone (2077/78–2081/82). Langtang National Park Office.

[ece372758-bib-0029] Måren, I. E. , and L. N. Sharma . 2018. “Managing Biodiversity: Impacts of Legal Protection in Mountain Forests of the Himalayas.” Forests 9, no. 8: 476. 10.3390/f9080476.

[ece372758-bib-0030] Matin, M. A. , V. S. Chitale , M. S. R. Murthy , K. Uddin , B. Bajracharya , and S. Pradhan . 2017. “Understanding Forest Fire Patterns and Risk in Nepal Using Remote Sensing, Geographic Information System and Historical Fire Data.” International Journal of Wildland Fire 26, no. 4: 276–286. 10.1071/WF16056.

[ece372758-bib-0031] McLauchlan, K. K. , P. E. Higuera , J. Miesel , et al. 2020. “Fire as a Fundamental Ecological Process: Research Advances and Frontiers.” Journal of Ecology 108, no. 5: 2047–2069. 10.1111/1365-2745.13403.

[ece372758-bib-0032] Mehmood, K. , S. A. Anees , S. Muhammad , et al. 2024. “Analyzing Vegetation Health Dynamics Across Seasons and Regions Through NDVI and Climatic Variables.” Scientific Reports 14, no. 1: 11775. 10.1038/s41598-024-62464-7. 38783048 PMC11116382

[ece372758-bib-0033] Mishra, B. , S. Panthi , S. Poudel , and B. R. Ghimire . 2023. “Forest Fire Pattern and Vulnerability Mapping Using Deep Learning in Nepal.” Fire Ecology 19, no. 1: 27. 10.1186/s42408-022-00162-3.

[ece372758-bib-0057] Mo, K. , Q. Chen , C. Chen , J. Zhang , L. Wang , and Z. Bao . 2019. “Spatiotemporal Variation of Correlation Between Vegetation Cover and Precipitation in an Arid Mountain‐Oasis River Basin in Northwest China.” Journal of Hydrology 574: 138–147. 10.1016/j.jhydrol.2019.04.044.

[ece372758-bib-0034] Ntinopoulos, N. , S. Sakellariou , O. Christopoulou , and A. Sfougaris . 2023. “Fusion of Remotely‐Sensed Fire‐Related Indices for Wildfire Prediction Through the Contribution of Artificial Intelligence.” Sustainability 15, no. 11: 9152. 10.3390/su151511527.

[ece372758-bib-0035] Nyeko‐Ogiramoi, P. , P. Willems , and G. Ngirane‐Katashaya . 2013. “Trend and Variability in Observed Hydrometeorological Extremes in the Lake Victoria Basin.” Journal of Hydrology 489: 56–73. 10.1016/j.jhydrol.2013.02.039.

[ece372758-bib-0036] Nyongesa, K. W. , C. Pucher , C. Poletti , and H. Vacik . 2023. “Evaluation of the Relationship Between Spatio‐Temporal Variability of Vegetation Condition Index (VCI), fire Occurrence and Burnt Area in Mount Kenya Forest Reserve and National Park.” Fire 6, no. 8: 282. 10.3390/fire6080282.

[ece372758-bib-0037] Pandey, H. P. , N. P. Pokhrel , P. Thapa , N. S. Paudel , and T. N. Maraseni . 2022. “Status and Practical Implications of Forest Fire Management in Nepal.” Journal of Forest and Livelihood 21, no. 1: 32–45. 10.3126/jfl.v21i1.56583.

[ece372758-bib-0038] Parajuli, A. , S. A. Manzoor , and M. Lukac . 2023. “Areas of the Terai Arc Landscape in Nepal at Risk of Forest Fire Identified by Fuzzy Analytic Hierarchy Process.” Environmental Development 45: 100810. 10.1016/j.envdev.2023.100810.

[ece372758-bib-0039] Paudel, A. , S. H. Markwith , K. Konchar , M. Shrestha , and S. K. Ghimire . 2020. “Anthropogenic Fire, Vegetation Structure and Ethnobotanical Uses in an Alpine Shrubland of Nepal's Himalaya.” International Journal of Wildland Fire 29, no. 3: 201–214. 10.1071/WF19098.

[ece372758-bib-0040] Peris‐Llopis, M. , B. Mola‐Yudego , F. Berninger , J. Garcia‐Gonzalo , and J. R. González‐Olabarria . 2024. “Impact of Species Composition on Fire‐Induced Stand Damage in Spanish Forests.” Scientific Reports 14, no. 1: 5229. 10.1038/s41598-024-59210-4. 38615154 PMC11016083

[ece372758-bib-0041] Pokharel, B. , S. Sharma , J. Stuivenvolt‐Allen , A. B. Shrestha , A. L. Westerling , and P. Thapa . 2023. “Amplified Drought Trends in Nepal Increase the Potential for Himalayan Wildfires.” Climatic Change 176: 17. 10.1007/s10584-023-03495-3.

[ece372758-bib-0042] Pokhrel, S. 2018. “Assessment of Above Ground Biomass and Fire Risk Zonation in Selected Forest Areas of LudhiKhola Watershed, Gorkha Nepal.” Remote Sensing of Land 2, no. 1: 47–64. 10.21523/gcj1.18020104.

[ece372758-bib-0043] Pokhrel, S. , and C. Sherpa . 2025. “Assessment of Spatiotemporal Variation of Vegetation and Its Relationship With Precipitation and Temperature in a Changing Climate in Nepal Mountain Ecosystems.” In Ecosystem‐Based Approaches for Resilience Building in Himalayan Landscapes. Disaster Resilience and Green Growth, edited by Y. I. Khan , M. Goswami , and S. Nautiyal . Springer. 10.1007/978-981-95-2007-7_6.

[ece372758-bib-0045] R Core Team . 2024. R: A Language and Environment for Statistical Computing. R Foundation for Statistical Computing.

[ece372758-bib-0046] Rawat, G. S. 1998. “Temperate and Alpine Grasslands of the Himalaya.” Parks 8, no. 3: 28–36.

[ece372758-bib-0047] Rogers, B. M. , J. K. Balch , S. J. Goetz , C. E. R. Lehmann , and M. Turetsky . 2020. “Focus on Changing Fire Regimes: Interactions With Climate, Ecosystems, and Society.” Environmental Research Letters 15, no. 3: 030201. 10.1088/1748-9326/ab6d3a.

[ece372758-bib-0048] Sanz, E. , A. Saa‐Requejo , C. H. Díaz‐Ambrona , A. Palos , and A. M. Tarquis . 2024. “Relationship Between Vegetation and Soil Moisture Anomalies Based on Remote Sensing Data: A Semiarid Rangeland Case.” Remote Sensing 16, no. 18: 3369. 10.3390/rs16183369.

[ece372758-bib-0050] Shrestha, B. , S. Wang , Y. Wang , H. Zhu , and W. Chen . 2024. “Spatiotemporal Patterns, Sustainability, and Primary Drivers of NDVI‐Derived Vegetation Dynamics (2003–2022) in Nepal.” Environmental Monitoring and Assessment 196, no. 7: 607. 10.1007/s10661-024-12754-4. 38858316

[ece372758-bib-0051] Tang, Q. , and G. Leng . 2022. “Vegetation Dynamics, Land Use and Ecological Risk in Response to NDVI and Climate Change in Nepal.” In Climate Risk and Sustainable Water Management. Cambridge University Press. 10.1017/9781108787291.009.

[ece372758-bib-0052] Tiwari, S. , N. S. Paudel , J. Sze , and R. Karki . 2022. “Unravelling the Local Dynamics of Increasing Fires in Community Forests of Mid‐Hills of Nepal.” Journal of Forest and Livelihood 21, no. 1: 60–71.

[ece372758-bib-0056] Tucker, C. J. , C. J. Tucker , J. E. D. Pinzón , et al. 2005. “An Extended AVHRR 8‐km NDVI Dataset Compatible With MODIS and SPOT Vegetation NDVI Data.” International Journal of Remote Sensing 26, no. 20: 4485–4498. 10.1080/01431160500168686.

[ece372758-bib-0053] Westerling, A. L. , H. G. Hidalgo , D. R. Cayan , and T. W. Swetnam . 2006. “Warming and Earlier Spring Increase Western U.S. Forest Wildfire Activity.” Science 313, no. 5789: 940–943. 10.1126/science.1128834. 16825536

[ece372758-bib-0054] Xiong, Q. , X. Luo , P. Liang , et al. 2020. “Fire From Policy, Human Interventions, or Biophysical Factors? Temporal–Spatial Patterns of Forest Fire in Southwestern China.” Forest Ecology and Management 474: 118381. 10.1016/j.foreco.2020.118381.

[ece372758-bib-0055] Zhang, Z. , B. Qi , G. He , et al. 2025. “High Resolution Global Forest Burned Area Changes Monitoring Using Landsat 7/8 Images.” Geo‐Spatial Information Science 28: 1–14. 10.1080/10095020.2025.2483429.

